# The Evolution and Future of Targeted Cancer Therapy: From Nanoparticles, Oncolytic Viruses, and Oncolytic Bacteria to the Treatment of Solid Tumors

**DOI:** 10.3390/nano11113018

**Published:** 2021-11-10

**Authors:** Kyle M. Pierce, William R. Miklavcic, Kyle P. Cook, Mikayla Sweitzer Hennen, Kenneth W. Bayles, Michael A. Hollingsworth, Amanda E. Brooks, Jessica E. Pullan, Kaitlin M. Dailey

**Affiliations:** 1 Biomedical Sciences, Rocky Vista University, Parker, CO 80130, USA; kyle.pierce@rvu.edu (K.M.P.); kyle.cook@rvu.edu (K.P.C.); mikayla.switzer@rvu.edu (M.S.H.); 2 Eppley Institute for Cancer Research, University of Nebraska Medical Center, Omaha, NE 68198, USA; william.miklavcic@unmc.edu (W.R.M.); mahollin@unmc.edu (M.A.H.); 3 Department of Pathology and Microbiology, University of Nebraska Medical Center, Omaha, NE 68198, USA; kbayles@unmc.edu; 4 Office of Research & Scholarly Activity, Rocky Vista University, Ivins, UT 84738, USA; abrooks@rvu.edu (A.E.B.); jessicaepullan@gmail.com (J.E.P.)

**Keywords:** nanoparticles, oncolytic viruses, oncolytic bacteria, exosomes, clinical trials, solid tumors

## Abstract

While many classes of chemotherapeutic agents exist to treat solid tumors, few can generate a lasting response without substantial off-target toxicity despite significant scientific advancements and investments. In this review, the paths of development for nanoparticles, oncolytic viruses, and oncolytic bacteria over the last 20 years of research towards clinical translation and acceptance as novel cancer therapeutics are compared. Novel nanoparticle, oncolytic virus, and oncolytic bacteria therapies all start with a common goal of accomplishing therapeutic drug activity or delivery to a specific site while avoiding off-target effects, with overlapping methodology between all three modalities. Indeed, the degree of overlap is substantial enough that breakthroughs in one therapeutic could have considerable implications on the progression of the other two. Each oncotherapeutic modality has accomplished clinical translation, successfully overcoming the potential pitfalls promising therapeutics face. However, once studies enter clinical trials, the data all but disappears, leaving pre-clinical researchers largely in the dark. Overall, the creativity, flexibility, and innovation of these modalities for solid tumor treatments are greatly encouraging, and usher in a new age of pharmaceutical development.

## 1. Introduction

Many cancer patients continue to experience grim prognoses in part due to treatment paradigms that can be as destructive as the disease they hope to address. Despite continuing improvements prompted by a deeper understanding of the underlying cellular mechanisms of cancer pathogenesis, the first generations of modern chemotherapeutics suffer from non-specific toxicity toward normal cells, leading to off-target effects. The treatment of tumor metastases is complicated further by the vast genotypic and phenotypic diversity often encountered, frequently within the same patient, and remains a challenge for researchers and clinicians alike. It is this newly recognized dimension of complexity that is, in part, driving the evolution of anticancer methodologies and the future direction of the field. Nanoparticles (NP), oncolytic viruses (OV), and oncolytic bacteria (OB) are multidisciplinary focal points that combine futuristic technologies ranging from genetic engineering and immunology to molecular pathophysiology and nanophysics. Here, a brief evolution of each modality within the broader field of oncotherapeutics is discussed, highlighting the future directions and intersections of each modality.

### The Unique and Challenging Context of Solid Tumors

The transition from normal, healthy cell to abnormal, tumorigenic cell occurs due to a series of genetic and epigenetic mutations, ultimately causing aberrant cell signaling pathways favoring immortality [1,2,3]. These characteristic mutations define the cellular interactions with the immediate environment [4]. Hence, any discussion of therapeutic approaches to cancer must necessarily consider the tumor microenvironment (TME), a substantial obstacle facing novel oncotherapeutic development. The TME, intimately connected with the core of solid tumors, consists of necrotic cells, hypoxic levels of oxygenation, and acidic pH levels, largely due to limited vascular supply. In addition, this environment has markedly abnormal immune regulation, giving rise to a niche of safety and immunologic privilege conducive to tumorigenic cell survival with limited to no immunologic interference [5,6]. Any successful therapeutic strategy must be capable of penetrating and surviving this harsh environment to be effective. 

Although the TME is an aberrant cellular microenvironment, it has its own homeostasis. As the tumor begins to grow, the vascular supply becomes relatively limited and abnormal, stimulating immature, disorganized angiogenesis through upregulated vascular endothelial growth factor/vascular endothelia growth factor receptor 2 (VEGF/VEGFR2) signaling, initiating activation of endothelial cells [7,8,9]. The change in vascularization leads to altered oxygen levels, dropping oxygenation and lowering the pH substantially [9,10]. Several mechanisms are in place to return to physiological oxygenation [11]; however, these mechanisms are largely short circuited in a tumor where oxygen saturation can be as low as 0.3–4.0% [12]. Interstitial pressure, calcification, and density of extracellular matrix (ECM) stroma, and baseline immune surveillance deviate from what is considered the physiological standard. In normal cells, the optimal activation of T cells occurs through the upregulation of CD40 and B7-1/2 on dendritic cells [13], but these signals are strongly inhibited by the TME. The TME produces a unique immunosuppressive environment with neoantigens, cytokines (e.g., TGFβ) and immune inhibitory cells (e.g., T-regs) that all work in concert to block normal T-cell signaling and create an immunologically privileged site for tumor proliferation [6,14,15].

While many question why an effective treatment for cancer has not yet been developed, the multifaceted way cancer attacks the body makes both drug design and selectivity delivery particularly difficult. Cancer cells hide in plain sight and are adapted to spread quickly, often remaining undetected until it is too late to intervene. These characteristics must be accounted for to provide alternative treatment strategies based on the type, stage, and location of the tumor. The ideal drug delivery system would have the capacity to distinguish and target tumorigenic cells—primary and metastatic alike—while leaving healthy cells unaffected. This oncotherapy thus must consider the route of administration, cellular signaling for precursors of metastasis, and the physiological effects after large-scale cell death in a relatively short time frame. Modalities such as nanoparticles, oncolytic viruses, and oncolytic bacteria provide a framework from which a unique solution can be derived, with the potential to target multiple tumor locations through the same treatment. In this review, we explore these three strategies through analysis of their advantages and pitfalls, while considering the future direction of these fields, which are more similar than they may seem at first glance. 

## 2. Nanoparticles

**Figure 1:**
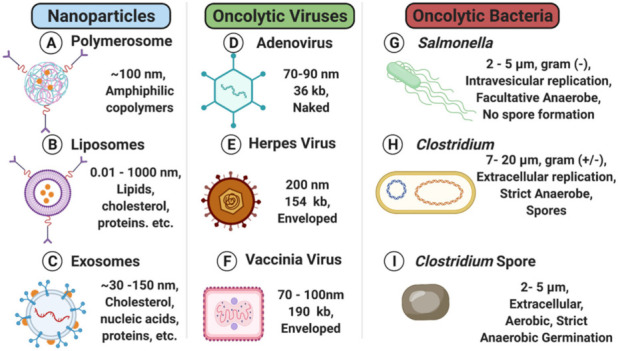
**Representative oncotherapeutic modality details, size comparison, and structural characteristics.** Nanoparticles: (**A**) polymersomes [21], (**B**) liposomes [22], and (**C**) exosomes [23]; oncolytic viruses: (**D**) adenovirus [24], (**E**) herpes virus [25], and (**F**) vaccinia virus [26]; (**G**) oncolytic bacteria: G. *Salmonella* [27], (**H**) vegetative Clostridium [28], and (**I**) *Clostridium* spore [28].

**Figure 2:**
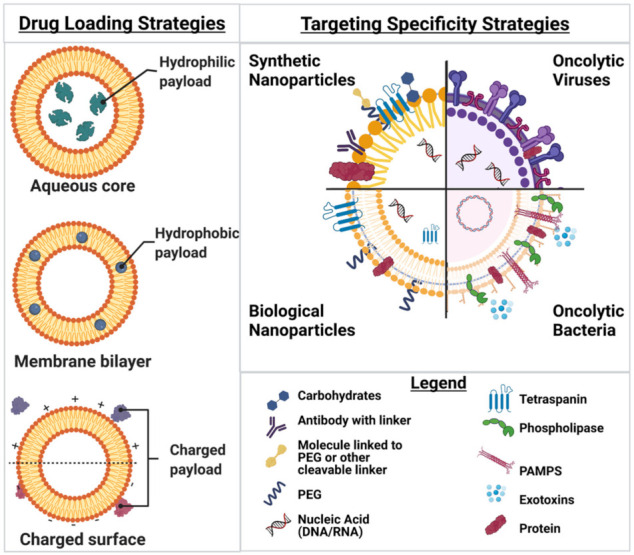
Representation of potential drug loading and targeting modifications strategies.

Nanoparticle (NP) drug delivery systems such as liposomes, polymersomes and exosomes (Figure 1A–C) have been in development for several decades with significant progress in a wide range of solid tumors. NP drug delivery systems facilitate directed delivery of a drug to the tumor, thus circumventing many of the off-target characteristics of current therapeutic options. The versatile nature of NPs allows for a vast combination of different materials, modifications, and payloads—an exciting prospect for the field. This versatility is due to nanoparticle building blocks that create both a modifiable surface and a customizable particle matrix [16]. To accomplish tumor delivery, nanoparticles take advantage of the enhanced permeability and retention effect (EPR), which allows for passive diffusion of particles less than 250 nm to localize to a tumor due to leaky blood vasculature associated with the TME and surrounding the tumor location [16,17]. The field of nanoparticle drug delivery contains a wide range of oncotherapeutic directions with various potential. This review makes important distinctions between liposomes, polymersomes and exosomes to provide context for the field at large, highlighting the most promising aspects for future development while keeping in mind that there are numerous in-depth reviews on each NP classification. Liposomes and polymersomes are synthetically based and can be relatively easy to manufacture with different chemical customizations (Figure 2). Exosomes are biologically based nanoparticles ubiquitously secreted by cells and therefore contain naturally synthesized biomacromolecules from their originating species. While exosomes have other advantages, customization can be difficult. Polymersomes, liposomes and exosomes are not the only nanoparticle formulations to focus on cancer drug delivery, though they are often the most prevalent; but it is worth noting self-assembled and inorganic nanoparticles are increasing in popularity, with several extensive reviews elsewhere [18,19,20]. Nanoparticle drug delivery systems have been used in many clinical trials. With several examples of successful clinical translation, pre-clinical studies continue to generate novel avenues for the delivery of complex payloads, increasing therapeutic concentrations and combating immune clearance prior to tumor localization. Each of these exemplar fields of nanoparticle studies, which are reviewed in greater depth below, have characteristic differences that can be exploited and utilized for novel oncotherapeutic generation.

### 2.1. Liposomes

Liposomes are lipid-based nanoparticles that mimic biological membranes in their basic lipid formation but differ from exosomes or polymersomes due to the lack of original markers [22,29] (Figure 1B). These lipid bilayer membranes have low permeability to hydrophilic drugs and high permeability to hydrophobic drugs such as Sorafenib and Tamoxifen [22,29,30,31]. Studies have since focused on stabilizing liposome hydrophobic drug payloads such as Paclitaxel with its highly potent broad spectrum of antitumor activity [32,33,34,35]. The specificity of the particle and/or drug release can be harnessed to modulate signaling cascades and stimulate the immune system, making liposomes both viable and highly specific [36]. In addition to multiple payload options, there are triggers and targeting motifs that can be utilized when designing liposomes to confer additional specificity. 

Some of these specificity modifications rely on the TME to deliver the drug payload. Environmental stressors, largely stemming from the solid tumor microenvironment, such as pH alterations, temperature, increased metabolite concentrations, and mechanical pressure have been utilized as endogenous environmental targeting modalities to trigger selective drug release [29,37,38,39,40]. For example, PEGylated, pH-sensitive, folate-coated, liposome-encapsulated Paclitaxel [39,40] contains both a targeting motif and release mechanism providing efficacy against metastatic breast cancer in in vitro studies [39]. Another recent study has suggested a new direction for the field by combining multiple areas of exploration: the newly developed metal-phenolic networks-integrated core-satellite nanosystem is a liposome combining encapsulated EDTA and membrane-bound near-infrared photothermal transducers [41]. The core satellite component is comprised of mesoporous silica nanoparticles encapsulating doxorubicin while simultaneously coated with a Cu^2+^-tannic acid metal-phenolic network [41]. This combination gave rise to selective payload release upon excitation of the near-infrared photothermal transducer, allowing for more explicit control. Positive outcomes of such an approach are indicated in in vivo studies [41]. This compilation of multiple targeting facets represents a potent future avenue for liposome design. 

The drawbacks of liposomes should be noted—one of which is the spontaneous fusion of liposome membranes, causing decreased drug payload concentration and increasing off-target toxicity [39,41,42]. The most common surface modification, PEGylation, was originally thought to increase circulation time, but additional research has since yielded several conflicting studies, complicating the utilization and implementation [43]. Alternatively, the addition of negatively charged moieties to the surface of liposomes has demonstrated both electrostatic repulsion and stabilization of the liposome, allowing effective drug delivery [41,44]. This avenue for liposome alteration generates a substantial increase in options for NP-hybrid drug delivery with characteristically high retention [41]. As with all drug delivery systems, liposomes have vast capacity if properly designed—keeping the innate immune system, biological barriers, and biochemistry at the forefront of development. 

### 2.2. Polymersomes

Polymersomes are a largely synthetic system composed of copolymer materials with characteristic alterations of hydrophilic and hydrophobic surface layers allowing for the development of tumor-specific targeting capacity (Figure 1A) [21]. These alternating hydrophobic properties lend themselves to surface manipulation, allowing for widespread differentiation and utilization (Figure 2) [21,45]. Release mechanisms are frequently incorporated into polymersomes, utilizing endogenous environmental conditions of the TME to elicit drug payload delivery. Hypoxia [46,47], pH, and temperature sensitivities have all been used with relative success and release triggering molecules typically conjugated to the base polymer [48]. 

Targeting the endogenous characteristics of the TME through polymersome conjugation has become a popular approach for chemotherapy delivery in refractory tumors [49]. An array of active targeting moieties, including ApoE [50,51], Arg-Gly-Asp (RGD) peptide [52,53,54,55], and transferrin [56], have been explored as avenues of modification [21,50,57], generating polymersomes selectively directed to tumor loci while minimizing toxicity [21]. RGD-modified poly-lactic-co-glycolic acid (PLGA) polymersomes loaded with Sorafenib and Quercetin demonstrated selective delivery to hepatocarcinoma cells with significant growth inhibition [52]. The addition of a chemosensitizer, such as Sorafenib, with the administration of chemotherapy takes advantage of distinct drug mechanisms and their synergistic actions [52], which are then further maximized by direct delivery to tumor cells [45,52]. This combinatorial therapy has gained popularity in pre-clinical research due to the synergy of specific drugs despite the potential for dosage issues when applied clinically. Alternatively, RGD, PEG and hyaluronic acid tagged polymersomes termed LightOn therapeutics, were successfully loaded with plasma DNA targeted to CD44 receptors [58,59]. Manipulation of LightOn transgene expression was used to modulate gene expression within the breast cancer TME, resulting in highly specific tumor inhibition and negligible off-target toxicity [58]. This strategy indicated a favorable avenue for the implementation of polymersomes, especially with the diverse and ever-evolving landscape of gene modification technology [58].

In addition to targeting cell surface markers, specific organelle targeting motifs have been implemented in pre-clinical experimentation. Targeting the nuclear pore complex with polymersomes may be a promising application; however, the channel transport mechanism for particles exceeding the pore diameter of 60 nm remains to be fully characterized, preventing large forward momentum in this field [60]. Nucleus specific polymersome binding via nuclear pore complexes has indicated potential, particularly for delivery of gene modification payloads [61]. Many gaps in knowledge remain for this technology, delaying both pre-clinical and clinical studies, including a noted delay in payload release within the nucleus, optimal surface interactions with nuclear pore complexes, and efficient nuclear uptake [61]. However, given the promise of gene modification as a disease state therapeutic or even cure, development of targeted polymersomes represents an interesting avenue of exploration.

### 2.3. Exosomes

Exosomes represent a unique avenue for oncotherapeutic delivery as they are not synthetically produced, but rather generated by membrane budding in eukaryotes (Figure 1C) [62]. Like liposomes, exosomes have a characteristic ability to bypass biological barriers as 30–150 nm extracellular vesicles. Exosome secretion has been documented by nearly every cell type with isolation possible from blood, urine, bovine milk [63], plants, and cell culture media [62,63,64,65]. Harnessing this naturally produced nanoparticle represents a relatively new field likely to impact both therapeutics and detection. The source of exosome isolation is a critical aspect of the design for this therapeutic as it directly impacts safety and scalability [23] and must be considered early in development. 

**Figure 3:**
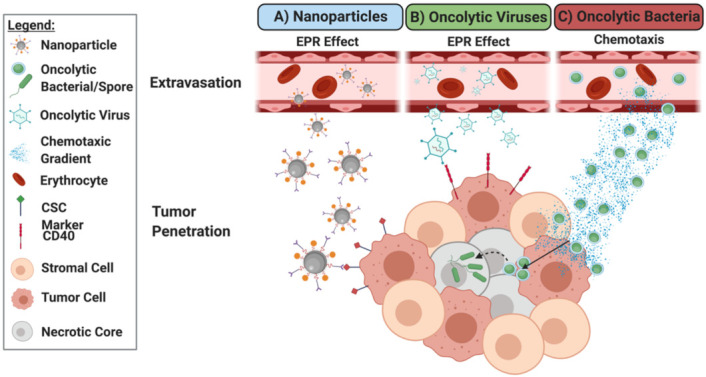
**Summary of tumor localization mechanisms.** (**A**) Nanoparticles use the Enhanced Permeability and Retention Effect (EPR) allowing molecules of less than 300 nm diameter to accumulate in tumor tissues due to abnormal tumor vasculature [17]. This figure depicts a generic nanoparticle targeting to a Cancer Stem Cell Marker (CSC) for entry and payload delivery; (**B**) Viruses also use the EPR effect in conjunction with upregulated cell surface markers for enhanced targeting specificity [68,69]. After entry the DNA or RNA payloads are delivered to the cell [70]; (**C**) Bacteria can follow chemokines to the site of the tumor before migrating to the hypoxic core to undergo sustained replication [71,72].

While exosomes play a prominent and growing role in diagnostics [64], they also provide an interesting mechanism for drug delivery (Figure 3A) [62]. Loading of doxorubicin into exosomes produced by immature dendritic cells engineered to express lysosome glycoproteins exhibited tumor targeting with evidence indicating efficacy against solid tumors [66]. Cell culture-derived exosomes were modified to incorporate anti-CD40 and anti-PD-L1, eliciting target specificity while encapsulating multiple immune stimulation drugs. The combination of several modifications indicated the in vivo potential by hindering tumor cell survival and metastasis through modification of immune response [67]. 

The ability to target exosomes and deliver a payload is clear from the data but modifying the content and the exosome bilayer is currently hampered by a lack of characterization. However, studies including modifications to the lipid bilayer and addition of targeting motifs as well as a variety of nucleic acid and protein cargos [23] are currently being conducted to increase retention time and targeting specificity (Figure 2). While the prevalence of exosomes as a method of targeted drug delivery is increasing, it is still in the relatively early stages [23]. The innate abilities of exosomes in cellular communication provide a method of exosome transportation within the body. An exhaustive characterization of innate exosome cargo has informed the development of nanoparticle materials to accomplish more sensitive payload delivery [62,63,64,65], but the process of identifying specific exosome components and subsequently accomplishing the translation of those components to other nanomaterials remains a substantial challenge. Unfortunately, use of exosomes is hindered by perceived safety, consistency, and scalability to accomplish clinical translation, especially as the mechanism for proliferation inside exosomes remains to be elucidated. Exosome-mediated cancer therapy could bridge the gap between multiple nanoparticle targeting strategies, generating significant growth and development for this relatively novel field. 

### 2.4. Advantages, Disadvantages, and the Future of Nanoparticle-Mediated Oncotherapy 

Nanoparticle biotechnology has accomplished clinical translation in vaccination and diagnostic technology, but efforts to accomplish direct oncotherapeutic application have experienced limited progress. Most nanoparticle targeting strategies, including material composition as well as targeting and triggering motifs, require surface presentation to the target tissue for efficacy. This has led to challenges for the field as different tissues exhibit biases in a variety of uptake mechanisms, and subsequently accept nanoparticle-mediated drug delivery with varying degrees of success. It is imperative to keep the intended target tissue characteristics in mind when developing novel nanoparticle-mediated therapeutics. Target accumulation of nanoparticles has also become a commonly experienced hurdle with multiple potential explanations, but more prominently premature clearance and non-specific binding/phagocytosis result in below therapeutic dosing with no efficacy. Furthermore, critical aspects of immune recognition, clearance, and non-specificity must be considered early in development. Moreover, while nanoparticle production is more conducive to replicability and scalability practices compared to the current state of oncolytic viruses and bacteria, attention to these details early in the development process will vastly improve clinical translation. Nanoparticle-mediated oncotherapy presents many advantageous characteristics with the potential to make current therapeutic methods more viable and effective by allowing both targeted and extended retention (Table 1). As with any novel therapeutics, perceived safety by both clinicians and society remains a looming challenge to accomplish clinical translation. Currently, the field is experiencing an influx of data, steadily addressing the knowledge gaps that hinder widespread clinical translation and acceptance, but it is undeniable that innovation and collaboration amongst similar fields such as oncolytic viruses and oncolytic bacteria are required to adequately treat the multitude of cancers still faced in the clinic. It is unlikely that a one size fits all approach will ever be successful.

## 3. Oncolytic Viruses

Oncolytic viral therapy utilizes genetically modified viruses capable of selective replication in tumor cells to mediate oncotherapy (Figure 1D–F) [24,25,70,73,74,75]. Unfortunately, early studies used unattenuated viruses with potent toxicities, almost ubiquitously resulting in severe—often fatal—adverse events [76], which not only halted on-going studies, but have had lasting impacts—stunting the development and translation of this technology [77]. Nevertheless, the advent of novel gene editing techniques has furthered understanding of viral biology, enhancing both safety and efficacy while renewing viral-based oncotherapeutic development [74]. The steps taken to accomplish clinical translation of oncolytic viruses are discussed as context for the field, highlighting mechanistic advantages and important modifications. 

### 3.1. Mechanisms of Oncotherapy

Antitumor activity characteristic of oncolytic viruses is thought to occur through two mechanisms of action: (1) selective replication within tumorigenic cells resulting in direct lysis and/or (2) induction of systemic antitumor immunity—notably, these mechanisms are not mutually exclusive [78]. Advancing knowledge has indicated host immune system activation is likely the most effective strategy [79,80]. Thus, as technological advances occur, acceptance of this therapeutic modality has grown significantly, and the field has begun to use modern techniques to customize oncolytic viruses, generating further specificity and efficacy (Table 1). 

As with nanoparticle-mediated delivery of oncotherapeutics, aberrant protein expression and subsequent signaling pathways result in targetable differences between normal and tumorigenic cells (Figure 3B) [68,69]. While some viruses, such as H1 autonomous replication viruses (e.g., parvovirus, reovirus, Newcastle Disease, etc.) have a natural preference for tumor cells [81], the majority of viruses can be adapted to provide tumor specificity. Oncolytic viruses have been engineered to maximize specificity by targeting upregulated surface marker expression [82,83,84], transcriptional elements unique to cancer cells [85,86,87], promotor or metabolic mediators [88,89], tumor specific defects in antiviral response [90], and combinations of such targets [91] (Figure 2). Pre-clinical and clinical models have highlighted the benefit of the enhanced oncolytic virus selectivity, which has limited viral toxicity [84,90,92]. These innovations provide the foundation for development of further modifications in pursuit of adequate selectivity and efficacy to accomplish clinical translation [93,94]. 

### 3.2. Combinatorial Oncolytic Viral Oncotherapies

Early studies uncovered an important limitation of oncolytic viruses: failure to generate significant immune response even after substantial tumor lysis [26,90,95,96]. This limitation was discovered through the combination of lysis with expression of representative tumor associated antigens (TAA), serving to focus the immune response [97,98]. However, the immune response was strongest towards the viral vector rather than to TAA [99,100]. Complicating this strategy further, the immune system developed significant quantities of neutralizing antibodies, resulting in limited repeated administration efficacy [101]. In fact, clinical trials have indicated that viral titer rapidly declines within a few days of intratumoral injection [78,102]. Thus, solely arming viruses with immunomodulatory mechanisms to generate a lasting antitumor response has proven largely unsuccessful with current technological capacities. However, oncolytic viruses could accomplish delivery of gene modification materials such as continuously expressed immunomodulatory transgenes [103]. 

Transgenes are coding sequences engineered to be expressed by oncolytic viruses (and bacteria) for the purpose of modulating cellular gene expression [95]. Examples of transgenes include: cytokines [70], chemokines [87], inhibitors of immune checkpoints [79,104], bi-specific T cell engagers [105,106], tumor antigens [107], and targets for chimeric antigen receptor T cells (CAR-T) [108,109]. Of particular promise is granulocyte–macrophage colony-stimulating factor (GM-CSF) [95,110]. GM-CSF is a pro-inflammatory cytokine known for increasing dendritic cell differentiation, recruitment and antigen presentation efficiency in tumor beds and draining lymphocytes [93,111,112]. Utilizing GM-CSF in clinical trials, Pexastimogene devacirepvec (Pexa-Vec or Vaccinia virus JX-594) [113] and Talimogene laherparepvec (T-VEC; Amgen) [80] have demonstrated effectiveness for coupling localized oncolysis with mediated immunomodulation [80]. Due to the successful outcomes of combinatorial therapy, new data are emerging regarding the benefit of coupling oncolytic viral therapy with immune checkpoint inhibitors, reversing TME immune suppression (Table 1) [114]. Tumors show an upregulation of expressed cytotoxic T-lymphocyte-associated antigen 4 (CTLA-4) responsible for downregulating T-cell activation and programmed cell death protein 1 (PD1), ultimately limiting T-cell effector functions and activities [114]. Utilization of the FDA-approved Ipilimumab, which enhances T cell priming by inhibiting CTLA-4 and subsequently reversing the negative feedback loop blocking dendritic cell stimulation [114] in combination with T-VEC not only had a tolerable safety profile, but the combination demonstrated greater efficacy than either T-VEC, Ipilimumab or Pembrolizumab alone [115,116,117,118]. Several oncolytic viruses are currently being evaluated for synergistic effects with chemotherapy, radiation therapy and other current oncotherapies [81,119,120,121,122].

### 3.3. Oncolytic Virus-Assisted Tumor-Imaging 

In oncology, the role of tumor imaging techniques (e.g., CT, MRI, PET and SPECT scans) is critical for diagnosis, staging and monitoring of new or recurrent tumors. However, current imaging modalities are relatively limited in their sensitivity, particularly for identifying very small or early-stage tumors [123,124,125,126,127,128,129]. Early detection of tumors can be directly correlated to patient outcomes, and thus represents a pivotal aspect of oncology that should not be ignored. Viral therapy can improve detection thresholds of these scans by engineering them with prodrug converting enzymes [130], receptors [131,132], or symporter/transporters [75,133] to facilitate deep tissue imaging [134]. The luciferase reporter gene in combination with the human Na+/I- symporter (*hNIS*) gene encoding sodium iodide symporter (NIS) has demonstrated transport of several other radioactive anions in addition to iodine, increasing the sensitivity of SPECT and PET imaging [135,136]. To date, oncolytic viruses have been engineered to express NIS with varying degrees of success [137,138,139,140,141,142,143], largely due to the challenge of increasing viral propagation to overcome the minimum threshold for detection [134,144]. Several theories have been proposed to understand this challenge, with emerging data indicating the TME can modulate NIS expression [133]. While further characterization is warranted, combined viral strategies are likely required in concert with viral imaging to maximize effectiveness.

### 3.4. Advantages, Disadvantages, and the Future of Oncolytic Virus Therapy

While each virus presents unique characteristics, an overarching theme has emerged: despite overwhelmingly favorable pre-clinical data, challenges related to potency, efficacy, tracking, and durable clinical responses have significantly hindered wide-spread progression through clinical trials [145]. Even with the success of T-VEC and Pex-c, therapeutic logistics such as direct delivery to tumors limit application to select tumor contexts. Oncolytic viral therapy would benefit strongly from improving the efficacy of systemic, intranasal, or oral administrations, thus both easing administration and broadening utility to detect, treat and prevent multiple tumor loci. While conceptually simple, realistically the presence of circulating antibodies [146] and the limited ability to achieve infiltration of dense tumor extracellular matrices (e.g., desmoplasia) as well as the necrosis present in solid tumor cores [147,148,149,150] limits systemic delivery capacity and may predispose the technology to acquired resistance due to incomplete tumor mitigation.

Studies have further demonstrated more than 95% of tumor gene mutations are unique and patient specific [151]; thus, broadly applicable targets are unlikely, limiting the use of this modality as a direct therapeutic. To accomplish direct targeting, each tumor presentation within an individual patient would need to be genotypically characterized, representing significant time and financial hurdles for clinical implementation, resulting in socioeconomic biasing for treatment availability. Furthering the socioeconomic divide, oncolytic viruses have shown the greatest effects when combined with costly immunotherapeutics. Finally, engineering of viruses is not only cumbersome in terms of manufacturing—limiting scalability and reproducibility—but requires significant investment in necessary biosafety measures and equipment for pre-clinical development that, given the limited applicability, may not be warranted in this context. However, oncolytic viruses are very promising as drug delivery modalities, particularly with recent CRISPR and RNAi advances. It is likely that this field will find applicability in gene modification oncotherapeutic delivery. The future remains hopeful for oncolytic viruses and the next decade with further technological advances may define viral oncotherapeutic utility.

## 4. Oncolytic Bacteria

Narratives of bacteria capable of tumor destruction date back to ancient Egypt, but the first clinical publication occurred in 1893 [152], providing tangible evidence of bacterial-mediated tumor regression. However, similar to early oncolytic virus studies, the inoculation of wild-type bacteria resulted in significant and intolerable toxicity (i.e., sepsis) [153], vastly curbing enthusiasm for further development. To overcome the toxicity of these treatments, heat inactivated strains *of S. pyrogens* and *Serratia marcescens* removed ‘toxins’ largely responsible for sepsis [154], greatly improving safety [27]—representing a critical step and renewing efforts towards clinical translation. With several decades of research and numerous safety studies now complete, oncolytic bacterial therapy has demonstrated safe and highly effective antitumor effects (Figure 1G–I). Several key species with prevalent engineering are briefly discussed for context, and their advantages along with remaining challenges for clinical translation are highlighted.

### 4.1. Oncolytic Bacteria: Attenuation and Mechanisms 

Perhaps the most critical paradigm for engineering oncolytic bacteria is reducing virulence without diminishing intrinsic antitumor activity [155,156,157]. Bacterial cells possess inherent pro-inflammatory, pathogen-associated molecular patterns (PAMPs), such as lipopolysaccharide (LPS), that elicit toll-like receptor (TLR)-family mediated stimulation (Figure 2) [158]. Modification of these potent immunostimulatory molecules must be harnessed to prevent systemic toxicity while still accomplishing antitumoral activities (Table 1). For example, during a simple heat-shock protocol, *Clostridium novyi* will lose the gene encoding α-toxin, which is primarily responsible for sepsis [159,160,161,162], while retaining its innate oncolytic capabilities. In contrast, *Salmonella* heat-shock attenuation resulted in minimal tumor regression and even a loss of colonization capacity entirely [28,163,164], demonstrating what can occur when the delicate balance between virulence and oncolytic capacity is upset [165]. To improve its safety profile, each oncolytic species must undergo specific and proven attenuation before any further modification is attempted. 

*Mycobacterium bovis* Bacille Calmette-Guerin (BCG), the first Federal Drug Adminstration (FDA) approved oncolytic bacteria [166], exerts antitumor activity by stimulating the release of inflammatory mediators CD-4, CD-8 and TNF-α, provoking a localized area of chronic inflammation to enhance immune surveillance and tumor regression [167]. *Salmonella* exhibits intrinsic oncolytic activity as an intracellularly replicating bacterium, while *Clostridium* secretes exotoxins and contains lipases on their surface to accomplish lysis. A number of mechanisms are proposed to underlie these oncolytic processes: nutrient deprivation [168], release of bacterial toxins [169], induction of counter regulation of intracellular pathways promoting autophagy [13], moderating antiangiogenic HIF-1α [170,171,172] and/or releasing nitrate reductase to promote apoptosis [173,174], with each particular species displaying its own characteristic effects. Studies of particular oncolytic bacteria have demonstrated the unique propensity to modify the local immune response in coordination with tumorigenic cell lysis [175], causing upregulation of pro-inflammatory cytokines and chemokines [126], increasing innate and adaptive immune cell infiltration to the TME [175,176,177]. T_reg_ cell concentration is thus decreased [169,178], subsequently transforming immunosuppressive myeloid-derived suppressor cells into TNF-α producing cells [179] and increasing concentrations of TAA on antigen presenting cells [180]. 

### 4.2. Targeting Safety, Delivery and Efficacy of Oncolytic Bacteria

**Figure 4:**
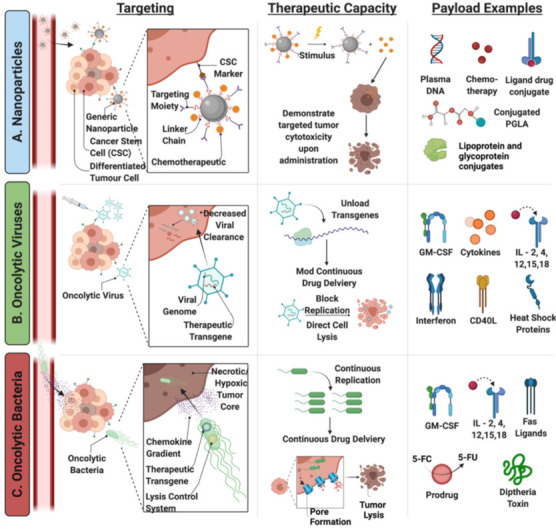
**Comparison of payload delivery characteristics and capacity.** (**A**) Nanoparticles use targeting motifs (e.g., cancer stem cell marker CSC) for specific targeting of tumor cells. Once localized, they will release their payloads with or without controlled stimuli [231,232]; (**B**) oncolytic viruses target tumors and take advantage of decreased viral clearance mechanisms. After they reach the cytosol, the virus will not only shed DNA/RNA transgenes resulting in constant replication, but they also block cellular replication or induce direct cell lysis. Examples of Oncolytic Viral payloads are depicted [70,102,104,144,146]; (**C**) Oncolytic bacteria migrate to tumor cells due to chemokine gradients. After reaching tumor cells oncolytic bacteria will either replicate within the tumor cell cytosol or further migrate to the hypoxic core before undergoing continuous replication and drug delivery. Examples of oncolytic bacteria drug delivery are shown for context [70,233,234,235,236].

The combination of hypoxia, pH, immune suppression, and the underlying abnormal vascularization makes drug delivery to the TME difficult for almost all oncotherapies. Intriguingly, these same characteristics provide the desired environmental niche for most oncolytic bacterial species (Figure 3C and Figure 4). Briefly, while the abnormal blood supply and lymphatics in tumors enhances the capture of bacteria [181], the bacteria simultaneously seek out tumors because of abundant nutrients [182,183,184,185]. Direct bacterial oncolysis enhances these effects as more nutrients are released from dead cells, creating a cycle of recruitment [186,187,188]. Both anaerobic and facultative anaerobic bacteria target the hypoxic tumor core for germination and survival [189,190], and the clearance of these bacteria once established is limited in part due to the immunosuppressive TME [191]. 

While details of each specific mechanism are explored in depth elsewhere [71,72,192], key aspects of *Clostridium* and *Salmonella* are included as promising representatives for the field. Unlike many spores that are considered dormant, *Clostridium novyi* spores are able to not only sense the germination conducive environment within the solid tumor core, but are also capable of migrating (based on a chemotactic gradient) to it. Once there, they are able to penetrate the desmoplasia from blood vasculature into the hypoxic tumor core, a difficult if not impossible task for most other chemotherapeutics, where they not only lyse the tumor, but act as a potent, life-long reactivation of the immune system against genetically similar tumors [28,191,193,194,195]. The potential of this particular species is furthered as the fully lytically capable, vegetative form cannot survive in any measurable level of oxygenation such as found in the blood stream or urine, and the spores, which are able to mitigate the toxic effects of oxygen, cannot initiate germination in any physiological level of oxygen, thus alleviating the risk of off-target events [162,187,189,194,196]. Improving the tumor localization capacity of *C. novyi* through CRISPR-mediated, stable genomic incorporation of an RGD peptide on the spore coat has recently been demonstrated feasible, indicating a promising new direction for customization to elicit better oncolytic capacity [157]. In contrast, *Salmonella* and *Listeria* species not only survive but proliferate in most intracellularly oxygenated environments, increasing the potential risk for off-target toxicity [196]. *Salmonella* use their flagellated membrane to migrate towards high nutrient concentrations produced primarily within the TME [182,183,184,185]. Removing metabolic genes, such as *pur*I, enhanced this effect through auxotrophy, which is the term for requiring specific metabolic intermediates, such as essential amino acids, from the environment in order to survive [197]. This auxotrophic strain accumulated at a tumor site 1000-fold more than in normal, non-tumor tissue—representing a significant gain in specificity [198]. Other strategies to increase oncolytic capacity include introduction of transgenes for surface receptors antibodies (e.g., epidermal growth factor receptor-2 and her2/neu [199]), antibodies against cell surface markers such as CD20 [200] and transient, plasmid encoded sequence for RGD [201], indicating modification of this species is not only possible but might also provide higher oncolytic efficacy.

### 4.3. The Optimization of Bacteria-Mediated Oncotherapeutic Payloads

Modifications regarding synthesis and delivery of anticancer payloads by bacteria are also worth briefly noting. The list of therapeutic payloads continues to grow, with focus placed largely on incorporation or secretion of cytokines, TAA, immune modulators, prodrugs, gene silencers or transport effectors [202,203] (Figure 4). For example, *Salmonella* avoids detection through use of a modified vacuole, the salmonella-containing vacuole (SCV), allowing time to accomplish replication—which could be exploited as a platform for either continuous drug delivery or to reach therapeutic levels higher than the initial dosage [204,205]. In an elegant study incorporating a deaminase gene capable of 5-FC to 5-FU conversion mediated by *Salmonella* for secretion inside tumor cells [206], resulting in a 3-fold increase in the 5-FU concentration. However, clinical translation stalled in phase I due to slow patient enrollment [207]. Further pre-clinical research into this strategy complicated this line of discovery when it produced evidence that certain synthesized payloads cannot cross both the bacterial envelopes and the SCV [208]. Thus, the development of both extracellular (e.g., autolysis [208,209,210,211], hypoxia [212,213,214]) and intracellular triggers (e.g., synchronized lysis circuit [215]), that improve timing and targeting as well as enhancing intratumor payload potency, have been generated. With a combination of payload and control systems, the effective delivery of oncotherapeutic drugs can increase tumor regression without significant adverse reactions.

Combination therapy of oncolytic bacteria and current chemotherapies indicated efficacy in early *C. novyi*-NT studies of a therapy termed COBALT, or combination bacteriolytic **t**herapy [161]. However, lack of methodology to address the current knowledge gaps of the *C. novyi* field hinders progress. *Salmonella* appears to enhance efficacy and safety of chemotherapeutics doxorubicin [216], cisplatin [217], gemcitabine [218], cyclophospho-amide [219], and combinations thereof (e.g., CHOP [220]). Radiotherapy associated oncolytic bacteria therapy has limitations because of toxicity to normal tissues as dose and frequency increase. While there is evidence of synergistic effects for the combination of oncolytic bacteria and radiation [221], the majority of benefits demonstrated are thought to be due to preferential colonization and immune modulation [222]; however, it is worth noting that combinatorial treatments have indicated efficacy above systemic administration alone [161] (e.g., polydomaine [223] and gold nanoparticles [224]), a possibility that should continue to be explored. Further work with *Salmonella* indicated PD-L1 and CTLA4 expression can be downregulated in a dose dependent manner [225,226], displaying oncolytic bacteria -mediated immune checkpoint inhibitor regulation, The intrinsic activity of bacteria may therefore replace, or at least minimize, the need for adjunct antibody-mediated immunotherapy—representing a distinct advantage over viral therapy. Pre-clinical testing of *S. typhimurium* in a murine model provided evidence that the bacteria was able to interfere with inhibitory receptor PD-1, enhance tumor regression, and prolong the survival rate of tumor-bearing mice [227,228]. While the understanding of bacterial interaction with checkpoint inhibitors has just begun, the prospective of these mechanisms warrants further investigation.

### 4.4. Advantage, Disadvantages, and the Future of Oncolytic Bacteria 

The natural ability of oncolytic bacteria to thrive within the hostile TME is a strong advantage over current chemotherapeutic strategies, but key challenges and concerns remain worth noting. First, the practical manufacturing, scalability, and reproducibility are of large concern for clinical implementation. As living organisms, unlike small molecules and other clinical agents, oncolytic bacteria cannot be sterilized through autoclaving or filtering—common methods of Good Manufacturing Practice (GMP)-grade drugs. Furthermore, the manufacturing of bacteria can be time consuming, depending on the strain and the supply chain. Current to writing this article, Merck & Co, the sole provider of BCG to the United States, has publicly stated they are experiencing a production shortage due to the challenging growth characteristics of the bacteria. While likely multifactorial, this shortage highlights how these critical aspects of oncolytic bacterial therapeutics must be accounted for early in pre-clinical development. Of additional concern is the fact that bacteria have the ability and propensity to undergo horizontal gene transfer [229] and are therefore prone to recombination, mutation, or plasmid loss prior to accomplishing tumor localization if alterations are not made to stabilize oncotherapeutic incorporation. This raises valid concerns regarding patient and public safety. Perhaps the largest hurdle to full clinical translation lies in public perception as it can quickly turn should safety or efficacy of these bacteria be inappropriately managed. While BCG has paved the wave for more bacteria to come to market, public perceptions regarding oncolytic bacteria, especially those formerly known to have pathogenic propensity, will always face an uphill battle. However, the application of genetic engineering technology represents a potent pathway to enhanced, stable safety and efficacy, lending support to a vast aptitude for oncolytic bacteria.

## 5. Comparing Nanoparticle, Oncolytic Virus and Oncolytic Bacteria: Development as Novel Oncotherapeutics

Novel nanoparticle, oncolytic virus, and oncolytic bacteria therapeutic developments all start with a common goal of accomplishing therapeutic drug activity or delivery to a specific site while avoiding off-target effects, whether that be leaching of the drug carried or unintended carrier activity. Typically, drug delivery design begins by selecting a specific target such as tumor location or tumor grade. Often, the specific, distinguishing characteristics (e.g., integrin display, microenvironment, immune status, etc.) of the target require analysis to determine the best delivery material—whether it be synthetic or biologic in nature. For example, pancreatic cancer, with its characteristic desmoplasia, poses several unique hurdles to drug delivery that must be accounted for during the design stages of novel oncotherapeutics [230]. In this circumstance, it would be advantageous to select a modality with the ability to actively penetrate this dense ECM—making oncolytic bacteria well suited for development of further therapeutic characteristics.

Once the best-suited system is selected for the intrinsic difficulties of the target tumor, it may seem that the pathway toward clinical trials varies drastically; however, there are many similar steps for all three modalities. Overall, there is a typical pathway that begins with genetic and physical characterization of the particle, then in vitro functionalization and validation, ending with in vivo small animal biodistribution and efficacy/non-inferiority studies. Each of these steps must occur before the true potential of the oncotherapeutic system can be determined. If the performance of the novel therapeutic is comparable to the current standard of care, then large animal in vivo studies are initiated, after which clinical trials proceed. In this section we break down each of these development phases for novel oncotherapeutic development to compare aspects of pre-clinical trial research and draw specific attention to the unique facets of each system, bearing in mind that each field would benefit from cross-contribution.

### 5.1. Generating Novel Therapeutics: Accomplishing Selective Targeting 

While perhaps obvious, identification of the disease state targeted by the therapeutic in development is a critical design step. Solid tumors have many similar physical characteristics (e.g., hypoxia, acidity), and yet there has not been a single therapy with widespread efficacy for multiple tumor targets. The characteristic differences between a hepatocarcinoma compared to a non-small-cell lung cancer are substantial and require consideration early in the design process. This includes selecting a relatively unique aspect of the specific tumor tissue for selective targeting to avoid damaging, off-target effects (Figure 4). There have been several types of targeting molecules that have been largely successful at conferring added specificity for novel therapeutics. These moieties can be grouped by their targeting strategy: cell surface, intracellular characteristics, endogenous environment, exogenous stimuli, and carrier cell-mediated delivery. 

#### 5.1.1. Cell Surface Molecules

Integrins represent fundamental regulatory components for many normal and abnormal cellular functions, including tumor initiation and metastases, as a result of their role in mediating cell adhesion and cell signal transport [237]. Many oncogenic mutations result in the dysregulation of the intracellular signaling pathways downstream of integrins, altering the surface expression of these integrin molecules. Combining this with the extensive body of literature characterizing the wide range of integrin functions in tumorigenic cells makes integrins a commonly selected target moiety [238]. There are twenty-four known integrin heterodimers, composed of 18 α-subunits and 8 β-subunits [238], each with its own unique preferential binding partners within the components of the extracellular matrix [239]. The complexity confers specificity, making integrins potent targets for selective therapeutic delivery. Interestingly, despite the complexity of the integrin dimer, their binding partners are relatively simplistic. For example, several integrins have been characterized to recognize a three amino acid residue of Arg-Gly-Asp (RGD) [240], which can be found in several extracellular matrix components. Various modifications to these peptides have been applied to add further selectivity and alter the intrinsic pharmacokinetics, with examples including cRGDfV [241], cRGDfK [242], RGD4C [243], and iRGD [244].

While arguably the most studied integrin-targeted ligand, RGD is not the only option to accomplish selective oncotherapeutic delivery. Asn-Gly-Arg, or NGR, is another integrin-binding motif derived from the integrin binding domain of fibronectin [245]. The NGR peptide structure has also been modified to produce several alternative motifs with selective integrin binding characteristics. Other short peptide sequences have indicated potential as well, for example, the integrin α_4_β_1_ recognizes the short peptides of EILDV and REDV originally identified from the larger peptide sequence of fibronectin. Recent work elucidated an ultra-selective tumor targeting peptide, α_v_β_6_-BP, that when conjugated to a fluorophore, identified a previously unknown metastatic tumor loci [246], providing evidence that such applications may go beyond therapeutics and include early detection. These simple recognition peptides are easily incorporated into nanoparticles and can be incorporated into oncolytic viruses and bacteria through genetic modification techniques. Thus, targeting integrins overexpressed or alternatively expressed on the surface of tumorigenic cells represents a feasible strategy for all three modalities of drug delivery discussed within this review [231,232,233,234,235,247,248,249,250,251].

#### 5.1.2. Intracellular Molecules 

The same cell signaling changes that precipitate and exacerbate the cell surface alterations allowing for integrin-mediated targeting also give rise to targetable intracellular alterations. Genomic mutations conferring advantages to tumorigenic cells often cause the loss of critical cellular defense mechanisms such as activation of Ras [252], overexpression of ICAM-1 [253], and suppression of interferon signaling pathways [254,255], making these cells uniquely vulnerable. One strategy employed to confer added specificity for oncolytic viruses included targeted mutations to amplify the interferon response [256]. Additionally, deletion of the RK3616 gene in the HSV-1 virus inhibited the downstream phosphatase, PKR (dsRNA induced protein kinase), making normal cells resistant to infection while leaving tumorigenic cells vulnerable due to disturbance of cellular antiviral pathways [257]. There are specific proteins with indicated tumor-type specificity: prostate specific antigen in prostate tumors, tyrosinase for melanomas, estrogen receptor protein and foetoprotein for hepatocarcinomas [258]. Each of these represents a potential unique target for oncotherapeutic delivery.

Essential genes for therapeutic cell infection could also be placed under the control of a selective promoter. Selective promoters can be identified by identifying overexpressed proteins. Human telomerase reverse transcriptase (hTERT), epithelial growth factor receptor, and survivin are commonly active and overexpressed proteins in a variety of tumor types [259]. The Wnt pathway contains several other proteins commonly implicated in multiple forms of tumors, particularly in the stomach and intestines. Adenoviruses have been modified to specifically target this pathway, though with mixed success [259,260]. Targeting the antiapoptotic pathways that characterize almost all tumorigenic cells has also proven a potentially promising strategy. One such study demonstrated that an E1B-19kDa gene deletion mutant enhanced cancer specificity through TNF pathways, significantly enhancing viral spread and antitumoral capacity while simultaneously maintaining selectivity [259].

By placing a gene critical for survival under the control of an inducible promoter corresponding to a selectively regulated protein, infection can have an added layer of tumor-specificity [198,235]. Several conditional mutations have been made to oncolytic adenoviral ability to replicate by deleting sections of the E1B protein, critical for replication processes through its suppression of p53 activity [260]. This has proven quite effective in bacterial studies regarding the extracellular environment in the form of programmed auxotrophy, though the efficacy of such a strategy post-infection has not been thoroughly studied [198,260,261,262]. It is worth noting the limited efficacy in oncolytic viruses as this strategy is largely restricted to DNA-based viral vectors. Nanoparticle use of this targeting strategy is also limited, as they represent less complex systems and are typically governed by cellular uptake through endocytosis rather than infection pathways. While this strategy is likely less directly effective for nanoparticle therapies, there are potential avenues to explore based on oncolytic bacterial design. Nevertheless, taking advantage of the many signaling differences that ultimately define and distinguish tumorigenic cells from normal cells represents a possible pathway to gain specific delivery for novel oncotherapeutics of all three modalities. 

#### 5.1.3. Endogenous Environment 

Perhaps the most ubiquitous aspects of solid tumors are those that result from the TME. These aspects include physiologically unique levels of hypoxia, acidity, and interstitial pressure—though none of these contexts are well defined or studied in situ of live, human tumors due to the difficulty maintaining such an environment while effectively probing characteristics. Utilization of these tumor aspects represents several advantages over the previously listed types of modification, notably selective targeting of physical tumor cell characteristics overcomes the heterogenous aspects intrinsic to solid tumors. 

**Figure 5:**
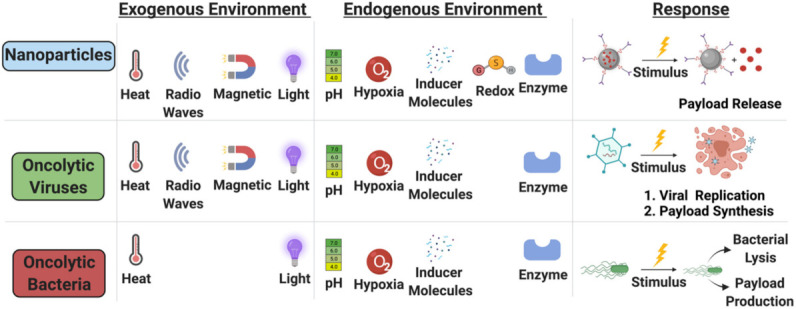
**Mechanisms to enhance drug delivery.** Examples of the exogenous and endogenous stimuli resulting in various drug or payload release. References—NP: [234,235,248], OV: [261,262]. OB [198,209,210,211,212,213,214,215,216].

Normal tissues have 2–9% oxygenation, while the hypoxia characteristic of the tumor core is significantly lower, ranging between 0.02–2% [12,263] (Figure 5). Hypoxia-activated nanoparticles are typically inactive in normal cells, and are activated in hypoxic cells or tissues when the modification undergoes reduction—typically catalyzed by oxidoreductases [236,263,264], including quinones [265,266,267], nitroimidizoles [268], aliphatic N-oxides [269,270,271], benzotriazine-N-oxides [272,273] and azobenzoic-oxides [274,275]), as well as transition metal therapies [276,277]. A hypoxia responsive promoter was effectively incorporated into the adenovirus *E1b* gene, preventing off-target expression of essential genes for oncolytic viral infection [278]. The vesicular stomatitis virus (VSV) is known to have a particular affinity for hypoxic environments [279]. Several oncolytic bacterial species have innate hypoxic sensitivities, especially those that are anaerobic [223,280]. It is worth noting that many of the moieties employed in hypoxia-responsive nanoparticles have the potential to be applied in various forms through genetic modification of oncolytic bacteria to confer added specificity [281].

Solid tumors are well characterized to be loci of high acidity in part due to the Warburg effect, where in tumorigenic cells have aberrant metabolism biased towards glycolysis with the byproduct of lactic acid exacerbated by inadequate lymphatic diffusion [282]. Similar to hypoxia exploitation, acidity can be targeted as well (Figure 5). Nanoparticles have demonstrated selectivity when modified with molecular moieties with pKa values near the tumor interstitial pH [282], allowing for the small pH drop within and near the tumor to trigger a conformational change in the functional group of the nanoparticle resulting in drug delivery [282]. Nanoparticles have utilized pH-sensitive groups (histidines, tertiary amines, and sulfonamides) [283,284], pH sensitive linkages [285] and pH-responsive insertion peptides featuring weak cellular membrane interactions at a neutral pH while capable of penetration and forming transmembrane complexes when triggered by pH [286]. Far fewer examples of oncolytic viruses targeting acidity exist, likely due to the vulnerabilities of viral particles when not contained within cells. However, one study probed an adenovirus coated with the pH-sensitive co-block polymer, PEGbPHF [287]. The pH-sensitive modified adenovirus had significantly higher antitumor activity upon systemic administration in animal models with xenograph tumors when compared to the non-modified adenovirus [287]. Another adenovirus modification employing the selectivity of acidity as a targeting strategy coated the virus with a pH-sensitive bio-reducible polymer, PPCBA [288], demonstrating feasibility of this mechanism. Again, as with hypoxia, the acidity targeting capacity of oncolytic bacteria is a naturally occurring proclivity of the species in question, but these innate characteristics could be bolstered through further genetic or chemical engineering [281].

#### 5.1.4. Exogenous Stimuli

Light, sound, temperature, radio frequencies and magnetic fields can also be utilized as external stimuli to release drug payloads carried on or within the modalities discussed in this review (Figure 5). These forms of stimuli represent promising avenues of specific payload delivery due to their non-invasive triggers. Radio frequency modulation has provided some evidence of efficacy, as have alternating magnetic field and photothermal, photodynamic and light activation stimulation. All these external stimuli function to generate hyperthermia eliciting a therapeutic release, with relatively successful applications in nanoparticle facilitated drug delivery [289,290,291,292]. Hyperthermic induction has also provided additional selectivity in oncolytic viral and bacterial directed infections. The combination of oncolytic herpes virus with hyperthermia increased viral growth by six-fold and resulted in lysis of approximately 80% of pancreatic cancer cells when infected [293]. Most bacterial species have optimal growth conditions of 37 °C, indicating that hyperthermic effects to reach these temperatures could lead to faster colonization and floridity of the tumor, ultimately resulting in more efficient lysis [291]. 

**Figure 6:**
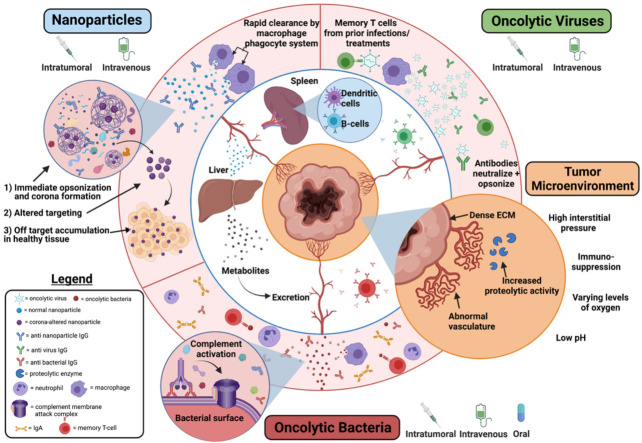
**Clearance and biological barriers to novel oncotherapies.** The outer ring depicts the initial interactions that occur for each therapeutic as it enters systemic circulation—including corona formation [249,303,304,305], innate immune responses [311,312], and adaptive immune responses [103,206,235,248,257,318]. Should treatments navigate these obstacles, the tumor microenvironment [133,146,150], metabolic pathways, and adaptive immune responses can complicate current and/or future treatments.

Both nanoparticles and oncolytic viruses face significant hurdles with environmental targeting selectivity due to the degenerative effects of the TME (Figure 6). The same challenges that affect intratumoral delivery of these modalities, especially availability of the tumor, also apply when utilizing exogenous stimuli. However, oncolytic bacteria have proven quite adept through both genetic engineering and innate mechanisms at effectively and selectively targeting the microenvironment at the core of almost all solid tumors (Table 1) [197,198]. Furthermore, oncolytic bacteria have benefited from auxotrophic modifications, utilizing the unique metabolic byproducts of the TME to incorporate multiple levels of selective targeting eliciting multilayered prevention of off-target effects [182]. 

#### 5.1.5. Carrier Cell-Mediated Selective Delivery

Oncolytic viruses in particular benefit from carrier cell-mediated delivery strategies as they rely almost solely on passive targeting to reach tumors when introduced systematically, though nanoparticles [32] and intracellular oncolytic bacteria [294] have also benefited from this approach. This strategy generates specific delivery while almost entirely bypassing pre-existing antiviral immunity [295]. While multiple studies focus on the cellular vehicles of the immune system, stem or endothelial cells are also options. Mesenchymal progenitor cells (MPCs) are easy to isolate, easy to propagate and easy to manipulate in the laboratory, making them potential cellular vehicles for any of the three therapeutic modalities discussed. When MPCs were infected with oncolytic adenoviruses, they demonstrated effective transport of the virus to the targeted tumors [296]. Studies are underway to probe the efficacy of bone-marrow derived cells to transport therapeutics to tumors as they are known to preferentially accumulate within tumorigenic cell populations [297]. Endothelial progenitor cells have also demonstrated migration through peripheral blood vessels effectively and selectively homing to tumor vasculature, with oncolytic measles virus accomplishing delivery to patient derived tumor mouse models [298]. 

Cancer cells themselves have been utilized as cellular vehicles, though largely in regional delivery studies. Tumor carrier cells were infected with oncolytic parvovirus and then inactivated through gamma irradiation, quite elegantly creating a microscopic “Trojan horse” capable of infecting tumors with oncolytic viruses [299,300], with the potential to localize to metastatic locations when introduced intravenously [301]. Tumorigenic cells are well characterized to affect the surrounding immune environments, including the potential to secrete immune cell recruitment chemokines [301]. It is possible to use these immune cells in a very similar manner to pathogenic infections—taking advantage of these innate cellular vehicles to further mediate specific delivery. CCL5, a tumor-derived chemokine has been detailed to actively attract CD4+, CD8+, as well as NK cells [81], with monocytes and macrophages known to extensively colonize solid tumors and potentially promote angiogenesis [255]. This activity could be considered both as a strategy for selective targeting a tumor and as an additional level of immune reactivation in the suppressed tumor microenvironment. Specific delivery of HSV-1, adenovirus, VSV, parvovirus, measles virus and vaccinia virus has been achieved by utilizing carrier cells [96]. 

### 5.2. Modification and Characterization of Novel Therapeutics 

Once the disease and its selective targeting aspect have been identified, several techniques can be employed to modify the drug delivery modality. Synthetic nanoparticles have a plethora of chemical reactions able to accomplish specific modifications. Nanoparticles, in large part, are restricted to chemical modification; oncolytic viruses and bacteria can make use of both this strategy and genetically based alterations. However, synthetic biology mechanisms can be applied to accomplish genetic modification of organisms to produce nanoparticles, especially exosomes. It is worth noting that most bacterial cell surfaces are charged; therefore, chemical modifications are generally relatively easy [248], nor is using biopolymers or enzymes secreted by oncolytic bacteria as indirect therapeutics [249].

After modification, each drug delivery modality requires specific characterization to confirm the physical changes enacted to improve the delivery system. Common techniques employed to confirm novel nanoparticle formulation include: nuclear magnetic resonance (NMR) spectroscopy, mass spectroscopy (MS), Western blot, immunofluorescent microscopy when antibodies are available, transmission electron microscopy (TEM) and variations thereof, atomic force microscopy (AFM), circular dichroism (CD), matrix assisted laser desorption ionization-time of flight mass spectrometry (MALDI-TOF MS), and proteomic analysis. Oncolytic bacteria and virus studies can employ many of the same methods, though genetic and proteomic methodology are higher in priority given the live biological characteristics inherent to such therapies.

After the initial physical characterization has been completed, in vitro functionalization studies must be done. It is important to note that genetic modification does not necessarily confer RNA or protein expression, nor does it ensure the functionality of the expressed moiety; thus, assays probing the performance of the incorporated targeting molecule such as adhesion assays or enzyme kinetic studies must be conducted prior to initiation of in vivo studies. Such characterizations can vary widely based on the type of moiety integrated and the type of carrier. Nanoparticle systems are often adequately characterized through cytotoxicity and drug release studies in monolayer tumorigenic specific cell culture. After an initial efficacy study in monolayer cell culture, many nanoparticle studies visualize particle internalization over time to ensure cellular uptake and probe the mechanism of action. However, monolayer cell culture methods lack many aspects of the tumor microenvironment—aspects that may be necessary not only for activating both selective targeting components of nanoparticles and biological targeting pathways of oncolytic viruses and bacteria, but also to fully appreciate the functional efficacy of the system in context. While monolayer culture studies can be informative when properly controlled, all three fields benefit greatly from studies that continue testing the potential of novel therapeutics in more complex in vitro models such as 3D spheroids or organoids that better represent the in vivo. For example, data regarding *C. novyi*-NT spores indicate that even in hypoxic conditions, monolayer cell culture was unable to replicate the bacteria’s in vivo lytic capacity [302], emphasizing the importance of considering the leap that each novel therapeutic must make from in vitro testing to in vivo deployment and highlighting the continued need for more in vivo like in vitro models during pre-clinical evaluation. Independent of the model used, it is paramount to confirm that the innate characteristics providing oncolytic capacity are not abolished or otherwise significantly affected by modification. While confirming the oncolytic character of the system after modification may seem intuitive, this characterization step is often impacted by the field’s limited knowledge of fundamental lytic processes. Proper controls must be meticulously employed, with attention being given, even at this early stage of development, to producing a consistent and scalable product. Inadequate attention to these critical factors has contributed to clinician hesitancy and failure to achieve clinical translation.

### 5.3. Establishing Biodistribution and Efficacy of Novel Therapeutic

After evaluation of the modified delivery system through in vitro studies to adequately characterize and establish functionality, in vivo studies, and the appropriate design of such studies, is the next critical step toward clinical translation. While the functional in vitro characterization of each modality is relatively unique, during in vivo testing, the modality is largely irrelevant. Unfortunately, this does not make in vivo experimental design much easier when making the jump from pre-clinical to clinical development. Over the last decade there has been an ever-increasing number of peer-reviewed publications regarding the application of these drug delivery systems; however, the full power of these tools is likely far from clinical translation. Multiple factors play into this gap between bench and bedside, but the hurdles encountered are markedly similar. Indeed, the degree of overlap is substantial enough that breakthroughs in one therapeutic could have considerable implications on the progression of the other two.

#### 5.3.1. Small Animal Model Selection

While no animal model can perfectly reflect the nuances of human disease states, selection of the best suited model system is largely determined by the hypothesis in question. Both the originating source of the tumor such as syngeneic versus transgenic tumorigenic cells and the selection of orthotopic, subcutaneous, or xenograph models of implantation as well as the host species—particularly the immune status—are important components for consideration. Current in vivo models are often limited due to either lack of a complete immune system or a biased immune system [53]. The evaluation of oncolytic viruses is further complicated as animal models frequently lack susceptibility [81]. Moreover, since these oncotherapies function in tandem with the immune system [43,250,251,281], selection of the appropriate pre-clinical murine model is a critical decision for clinical translation. Immune cell populations are altered due to tumors, pre-existing disease states, and previous treatments—which can increase clearance and usually are not replicated in pre-clinical animal modeling [303]. Most healthy humans have a balanced Th-1/Th-2 response [43,251,304]; therefore, both Th-1 and Th-2 biased models, which includes many of the most common, wild-type murine strains, should be considered. However, it is worth noting direct comparison of clearance concluded that Th-2 biased mice are the most stringent when determining in vivo clearance [304].

Oncolytic viruses and bacteria can elicit significant immunogenic response as the host immune system is designed to mitigate infection, often adding difficulty, time, and cost to the initiation of in vivo studies due to concerns regarding safety, toxicity, and biocontainment. These valid concerns require attention to stringent laboratory conditions and protocols to protect research personnel and public safety, despite the advances of attenuation. The requirement to have and run an adequate biosafety environment for experimentation, as well as the training required to work safely, can be a significant hurdle for these fields.

#### 5.3.2. Immune Clearance and Biological Barriers

Perhaps the most important consideration for in vivo testing of NPs, OVs, or OBs is protein corona formation and immune clearance capacity (Figure 6) [305]. Once a drug delivery modality enters the bloodstream, it is quickly and inevitably coated with opsonizing proteins from circulation to form a protein corona [43,306]. Increased clearance by the macrophage phagocytic system (MPS) is then initiated due to aspects of the corona, possibly provoking safety issues and off-target effects [251,306,307,308]. Protein corona formation is variable depending on the biological environment [307] and can lead to issues with targeting and drug release [309]. To account for opsonization, incubation of nanoparticles in serum prior to observing cell interactions has been explored [303,310,311]. These pre-incubation studies demonstrate that corona formation differs substantially between species [311]; thus, it has been suggested that therapeutic molecules should be incubated in plasma from the intended animal model [303]. PEGylation, surface-linked albumin, and other surface modifications attempt to evade corona formation; however, studies have found that some patients quickly developed antibodies against these modifications upon repeated treatments, drastically increasing clearance [312,313]. Anticipating corona formation and its consequences on therapeutic targeting and metabolism is critical to development of safe and effective therapeutics. 

While the protein corona can provoke opsonization, microbial specific clearance can occur through a variety of mechanisms, primarily binding complement proteins to PAMPs, initiating a cascade leading to phagocytic clearance (Figure 6) [314,315]. A variety of other clearance mechanisms are present in the blood stream and tissue, which promote the rapid clearance of oncotherapeutic microbes including defensins, mucosal IgA, and circulating macrophages [316]. This variety must be considered. Even after oncolytic viral or bacterial infiltration of target cells, they must still evade immune detection by intracellular mechanisms such as RIG-1, a cytosolic receptor that recognizes and binds potential pathogens, eliciting a severe immune response and apoptosis of the infected cell [317,318]. Immune clearance represents one of the most substantial hindrances to successful oncotherapy and will continue to be, although harnessing these characteristics in a cell-mediated delivery manner represents a very promising strategy.

#### 5.3.3. Route of Administration

Administration route is critically important to all modalities of oncotherapeutic delivery as choosing the most tacit route of administration directly impacts clinical translation, and thus, should be considered from the conception of a novel therapeutic. At present, the majority of nanoparticles reach the target passively via the EPR effect [17], thus, allowing for relatively specific delivery through intravenous (IV) infusion. The IV route has also gained popularity for microbial based treatments due to their ability to directly seek out and target both primary tumors and their metastases [319,320]. Like nanoparticles, IV-administered oncolytic viruses and bacteria must contend with both innate and adaptive immune responses to reach tumors [106,206,250,321]—a new version of the “race for the surface”. Currently, the intratumoral route has had better therapeutic outcomes from oncolytic viral therapy, largely due to poor perfusion of the viruses into dense tumors (Figure 6) [322,323]. However, the intratumoral route is notably more complex than an IV infusion as it is generally performed under ultrasound or CT guidance, adding layers of clinical complexity to this administration [324,325]. Improved IV therapy has been attempted for oncolytic viruses and nanoparticles through addition of ECM digesting enzymes [149,326,327,328] to physically counteract the effects of the TME, among other modifications. However, there is concern these same mechanisms could potentiate metastasis, as has become evident with other chemotherapies [329]. Systemic administration of oncolytic viruses or bacteria raise the question of replication and damage in normal cells, but this is mostly unfounded with little to no literature evidence [69,330].

### 5.4. Large Animal Models and Clinical Trial Initiation

At the conclusion of successful in vitro and small animal studies, large animal studies, typically utilizing primates, but sometimes canine or porcine models, must be implemented prior to initiation of clinical trials. While testing of novel therapeutics in these models is critical to progression towards clinical translation, many have difficulty reaching this stage. Facilities, funding, and proper training are among the largest hindrances for development beyond small animal models. Larger institutions or private companies are often required to handle this next step toward clinical translation as smaller—particularly academic—institutions and companies are ill-equipped to pursue promising therapeutic development, pushing into large animal models. Fortunately, collaborations amongst varying sizes of organizations both academic, private, and even governmental are becoming more common—representing a critical step in a more fruitful direction. Overall, each modality for generating novel therapeutics has specific characteristic advantages and disadvantages. These aspects depend in large part on expertise, facilities, and innovation. While the oncotherapeutic capacity of nanoparticles, viruses, and bacteria seem drastically different, they have surprisingly similar characteristics and patterns of development. It is highly likely solutions for the challenges faced by one modality will be found in the innovation derived for another modality, making communication and collaboration critical to the shared goal of generating a selective but effective oncotherapeutic.

## 6. Overview of Clinical Trials

Progression of nanoparticle, oncolytic virus, and oncolytic bacteria technology into clinical application has been hallmarked by interplay between the fields in which breakthroughs in one technology impact the development of the others (Figure 6). Tumor localizing peptides, RNAi, and CRISPR-mediated gene editing have all been implemented as successful modification techniques; however, there are discrepancies between the dates of implementation. A 15-year gap exists between application of tumor specific peptides in nanoparticles and when this approach was applied to oncolytic bacteria. Similarly, it took several years from the first studies of RNAi effects on nanoparticle therapeutics before this method was applied in oncolytic viruses or bacteria. This lack of cross disciplinary communication and collaboration has likely strongly contributed to stagnated development over time. To bring these similarities to the forefront of the field, significant clinical trials and therapeutic trends are highlighted with discussion of pivotal FDA-approved therapies from each modality.

### 6.1. Nanoparticle Oncotherapeutic Trials

Despite ever-increasing pre-clinical publications regarding the development of novel nanoparticle oncotherapies, relatively few have progressed into clinical trials. A search of PubMed reveals that since 2010, over 43,000 articles discussing “nanoparticles” and “cancer” have been published, but only about 230 (~0.5%) discuss clinical trial results. Considering the limited amount of human research being conducted, it is unsurprising to note only 3 new nanoparticle drugs have received FDA approval in the last decade [290]. This is particularly concerning given the many advantages possible with nanoparticles. 

**Figure 7:**
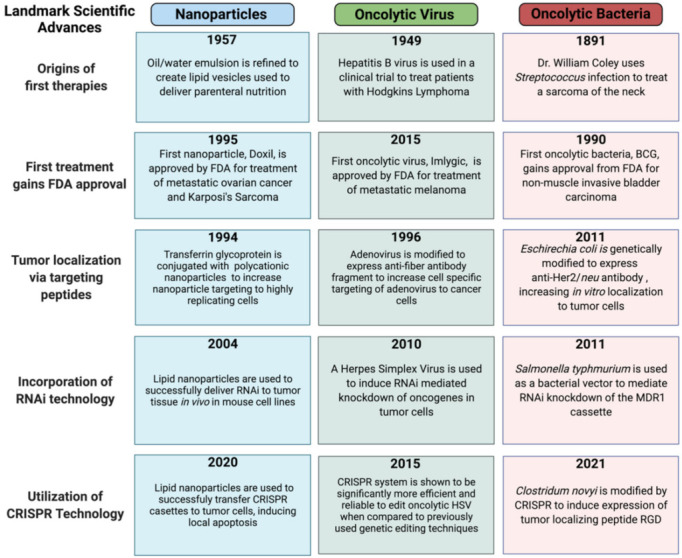
Significant milestones for the development of nanoparticles, oncolytic viruses, and oncolytic bacteria as oncotherapies. References—NP: [344,346,361]. OV: [74,361,362,363,364,365]. OB: [157,204,366,367,368].

The first FDA-approved oncotherapeutic nanoparticle, Doxil, gained acceptance in 1995 for the treatment of AIDS-related Kaposi sarcoma (Figure 7). Doxil is a PEGylated liposome encapsulating the chemotherapeutic doxorubicin. Application of doxorubicin in this manner dramatically reduced associated toxicities while increasing the localization of the drug to the tumor site [331,332]. Abraxane, the protein-based nanoparticle delivering paclitaxel for solid tumor treatment, followed with its approval 10 years later [333,334,335,336]. The accomplishment of clinical translation for these therapeutics effectively paved the way for the development of other nanoparticle oncotherapies [32,290,337,338].

Since the clinical implementation of Doxil and Abraxane, nanoparticle based systems have been explored in clinical trials due to their ability to deliver a vast array of payloads including gene therapy [339,340], cytokine mRNA [341], saRNA [342], microRNA [343,344], siRNA [345,346], and chemotherapy [338,347,348]. Liposomes have continually reaffirmed efficacy as clinically tolerable frameworks, fine-tuned by surface modifications to improve accuracy and efficacy while simultaneously limiting off-target effects [349]. For this reason, of the twelve currently approved nanoparticle oncotherapies, eight are liposome-based formulations [350]. Immunoliposomes, a variation of the successful liposome framework, are created by tethering tumor specific antibodies to a liposome to add target specificity, have advanced through phase I clinical trials [351].

Current clinical trials for exosomes have focused application to biomarker analysis and diagnostics [232,352,353,354]. IFN-γ-dendritic cell-derived exosomes, for example, were loaded with MHC class I- and class II- restricted cancer antigens with a demonstrated ability to halt progression of non-small-cell lung cancer in a phase II clinical trial [355], indicating the capacity of dendritic cell-derived exosomes to boost the natural killer and T cell antitumor functions. Pre-clinical models are searching for additional immunotherapeutic applications such as inducing cross-linking between T cells and EGFR-expressing breast cancer cells [356], treating with cytotoxic exosomes derived from chimeric antigen receptor T cells [357], and improving cancer vaccine efficacy [358,359]. Use of exosomes whether dendritic cell- or bovine milk-derived, for drug delivery is striking and likely to be incorporated into clinical trials soon due to many shared features with liposomes. However, a concern in the field remains regarding purification and quality assurance processes, which need improvement before exosomes can be responsibly applied at the clinical scale [62]. Nevertheless, several phase I trials are underway using exosomes for delivery of complex substances, such as siRNA [360], with many more likely to follow.

### 6.2. Oncolytic Virus Clinical Trials

Oncolytic viruses are the latest modality to gain official FDA approval (Figure 7) [369]. In 2015—20 years after the first nanoparticle and 25 years after the first approved oncolytic bacteria—Imlygic was ushered in as the first FDA-approved oncolytic virus. Imlygic is a genetically engineered strain of HSV-1 targeting tumorigenic cells through interactions with surface-bound nectins and selectively replicating only in the presence of disrupted protein kinase R and type I interferon pathways [370]. As it replicates within tumorigenic tissue, it also induces the expression of GM-CSF by the cancer cells, a cytokine that promotes the differentiation of white blood cells and facilitates further local immune activation [320,369,370,371,372]. This combination of engineered immunostimulatory abilities alongside a virus’s innate oncolytic properties has proven to be a powerful oncotherapeutic tool—worthy of incorporation in other novel therapeutics. Reolysin is another example of an FDA-approved oncolytic virus, as an unmodified reovirus displaying significant innate oncolytic and immunostimulatory properties, resulting in fast-tracked approval in 2017. Modifications of this successful oncolytic virus are already underway to incorporate *GM-CSF* genes into the genome [372].

Despite their vast potential, oncolytic viruses have had limited success as a monotherapy. As discussed previously, this is largely due to natural immunological barriers, tumor microenvironments, and tumor physiochemical properties [373]. Immunotherapy and virotherapy augment each other, leading to the popularity of combinatorial therapeutics [253,374]. V937 (Coxsackievirus A21), which illustrates this strong combinatorial approach, is under investigation for the treatment of patients with stage IIIc-IV melanoma. With an ability to increase CD8+ T cell activation and PD-L1 expression in the TME [250], V937 synergizes powerfully with checkpoint inhibitors, enabling T cell migration and infiltration into tumor loci [250,362,374].

Clinical progression must delicately balance immune suppression to allow viral entry and replication with ensuring an immune response once viruses infect tumor cells, specifically if the virus has an immunostimulant payload [303,364,365]. Immunotherapy, host immunity, and virotherapy are potently synergistic oncotherapies and successful treatment will likely hinge upon combining their strengths [303,321,361,362,363,364,365,370,371,372,373,374,375,376,377].

### 6.3. Clinical Trials of Oncolytic Bacteria

Only a few select oncolytic bacteria have progressed to clinical trials with only one gaining FDA approval [281,294,378,379]. BCG is an attenuated strain of *Mycobacterium* with tremendous value as a treatment for non-muscle invasive bladder carcinoma. In 1990, BCG was approved by the FDA and has been the standard of care since (Figure 7) [379]. BCG therapy works via catheter injection of a BCG solution into the patient’s bladder, with the mechanism of action largely unknown. However, it is known that direct contact with the malignant tissue is required to induce a cytotoxic effect and inflammatory response [380]. Despite the early success of BCG, significantly few oncolytic bacteria have made it to clinical trials since—especially when compared to nanoparticles and oncolytic viruses. Three species are currently at the forefront of clinical translation: *Clostridium novyi*-NT, *Salmonella typhimurium*, and *Listeria monocytogenes*. These species share similar features with other oncolytic viruses and bacteria, including pre-clinical genetic engineering, demonstrated antitumor effects in animal models, and natural or artificially enhanced tumor targeting capabilities. However, each of these species displays markedly distinct oncolytic mechanisms [157,201,281,323,366,367,381,382,383].

*L. monocytogenes* (ADXS11-001) is poised to reach FDA approval first. It is currently being investigated in a phase III clinical trial for the treatment of cervical cancer (NCT02853604). In stark contrast to the immune evasion commonly sought by other therapies, *L. monocytogenes* was designed to be phagocytized by antigen-presenting cells and secrete an antigen-adjuvant fusion protein to alter the TME [368], facilitating T-cell infiltration and reducing the inherent immune suppression characteristic of the TME [368,384,385]. This unique example highlights how the immunogenicity of an oncolytic bacteria platform can be harnessed successfully. *C. novyi*-NT has completed a phase Ib clinical trial (NCT01924689) and is well-tolerated in solid tumor patients, which is a considerable advancement for the field [384]. Currently, *C. novyi*-NT intratumoral injections are entering a phase II clinical trial while simultaneously being investigated in a phase I trial as a combinatorial therapy with anti-PD1 antibody, pembrolizumab [381]. Pre-clinical data indicate that *C. novyi*-NT combination bacteriolytic therapy (COBALT) has potent anticancer efficacy due to the contrasting cytotoxicity mechanisms and extremely selective innate targeting [161]. *S. typhimurium* is also progressing through the clinical trial pipeline. At the forefront of *S. typhimurium* research is the strain called Saltikva, which in addition to the knockout of virulence factors, has been engineered to induce IL-2 gene expression in the TME [258]. Pairing of these oncolytic bacteria with an immunostimulatory cytokine is a consistent trend in the field [386], as already demonstrated with aforementioned Imlygic and Reolysin. A phase II clinical trial for patients with metastatic pancreatic cancer is currently in progress [387]. 

### 6.4. The State of Nanoparticle, Oncolytic Virus, and Oncolytic Bacteria Clinical Progression

Understanding the mechanisms behind successful clinical translation is critical to provide pre-clinical direction, yet the recurrent pitfalls plaguing pre-clinical and clinical trials are far from transparent as the lack of negative information regarding why clinical trials fail limits progress. Numerous abstracts and publications report the efficacy of new and exciting oncotherapies, but when these therapies go into clinical trials, they seemingly vanish with no report of what went wrong. From a systematic review of the clinicaltrials.gov database, 177 of the 609 clinical trials were filed as complete; however, only 41 posted results to the database. The remaining 136 clinical trials had scant to no information on why the trial was concluded or any information about the results of the trial. With a glaring 76% of clinical trials not reporting results, scientific process is crippled, committing researchers to a futile cycle of repeating doomed strategies, wasting time and resources. Negative data can be as useful in this context as positive data to guide the field forward. For research in novel oncotherapeutics to continue its evolution to meet the ever-growing need for effective oncotherapies, a more transparent process must be developed in order to ensure that proper reporting is accessible for all.

**Figure 8:**
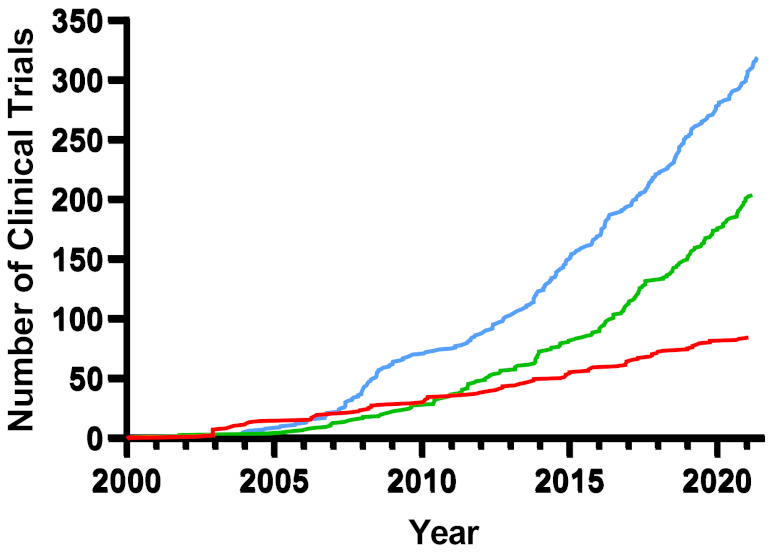
Running total of the number of clinical trials published since 1 March 2000 that investigated NP, OV, or OB as cancer treatments in phase I–IV clinical trials. Between 1 March 2000 and 1 September 2021, 321 total clinical trials related to NP (blue) treating cancers were published; 203 total clinical trials related to OV (green) treating cancers were published; and 85 total clinical trials for OB (red) treating cancers were published.

Furthermore, though there are similar strategies and methods implemented in the development of all three modalities, as has been noted several times in this review, a sharp discrepancy can be observed between the rate and total number of clinical trials published investigating each therapy. An in-depth search of the US clinical trials database was performed. Through a series of targeted searches an extensive, though not exhaustive, list of all clinical trials published since 2000 that used OV, OB, or NP therapies to target cancers was assembled. After collection of all clinical trials (609) that related to the relevant search terms, the trials were individually appraised to determine a variety of metrics to include: search term, tumor-localizing treatments, dates published, results published, completion status, target cancer. The dates that these clinical trials were first published were then plotted on a graph over time (Figure 8) to show the cumulative number of clinical trials that were published at any given date since 1 March 2000. Nanoparticle trials clearly surpass the other therapies, garnering the most interest in the past two decades, with oncolytic viruses being a clear second, and oncolytic bacteria trailing significantly behind. The reasoning for this discrepancy in clinical trials is likely due to many factors such as cost, ease of access, and level of scientific interest. However, the development of new techniques many level the playing field in the near future.

## 7. Conclusions

The introduction of targeted drug delivery modalities in oncotherapy has the potential to minimize cell damage extraneous to the tumor that is commonly encountered with conventional therapeutics. Several strategies are employable in nanoparticles, oncolytic viruses, and oncolytic bacteria to confer added selectivity and efficacy, with much of the pre-clinical development using overlapping methodology, indicating that these fields would strongly benefit from collaboration and communication. However, all fields have been slow to reach clinical trial initiation, with a particular bias towards nanoparticle research. Once studies enter clinical trials, the data all but disappears, leaving pre-clinical researchers in the dark regarding the best ways to evolve these oncotherapeutic modalities. In efforts to develop novel oncotherapeutics, negative data regarding why a therapy failed clinical trials can be just as important as positive data. Overall, the creativity, flexibility and innovation of these fields are greatly encouraging, making it likely that it is no longer a matter of *if* cancer can be cured, but rather *when* cancer will be cured, ushering in a new age of pharmaceutical development. 

## Figures and Tables

**Table 1:** **A comparison of delivery systems for OB, OVs, liposomes, polymersomes and exosomes.** This compares the difference in structure, proliferation in tumors, opportunity for genetic modification, tumor targeting, drug delivery capacity, immunomodulation, and anticancer effects and is a synthesis of the information contained in Section 2, Section 3 and Section 4 of this review.

Therapeutic Aspects	Liposomes	Polymersomes	Exosomes	Oncolytic Virus	Oncolytic Bacteria
**Structure**	Lipid bilayer membrane	Lipid bilayer membrane	Lipid bilayer membrane	Nucleocapsid	Cellular
**Proliferation in tumors**	No	No	No	Yes	Yes
**Genetic Modification**	N/A	N/A	N/A	Good	Good
**Tumor Targeting**	Specific and modifiable	Specific and modifiable	Specific and modifiable	Intratumor injection preferred to increase efficacy	Specific with systemic injection
**Drug Delivery capacity**	Contained within an aqueous core	Contained within an aqueous core	Contained within an aqueous core	Limited capacity of continuous expression	Continuous drug expression with termination control mechanisms
**Immunomodulation**	Low-Mild	Low-Mild	Low-Mild	Mild-Mod	Strong
**Anticancer Effects**	Drug delivery	Drug delivery	Drug delivery	Direct: cellular lysis Indirect: gene delivery and drug delivery	Direct: exotoxin and nutrient competition Indirect: unlimited delivery options

## Data Availability

Not applicable.
